# Generalisability of and lessons learned from a mixed-methods study conducted in three low- and middle-income countries to identify care pathways for atrial fibrillation

**DOI:** 10.1080/16549716.2023.2231763

**Published:** 2023-07-19

**Authors:** Tiffany E Gooden, Jingya Wang, Alessandra Carvalho Goulart, Ana C Varella, Meihui Tai, Vethanayagan Antony Sheron, Hao Wang, Hui Zhang, Jiaoyue Zhong, Balachandran Kumarendran, Krishnarajah Nirantharakumar, Rajendra Surenthirakumaran, Isabela M Bensenor, Yutao Guo, Gregory Y H Lip, G Neil Thomas, Semira Manaseki-Holland, On behalf of the NIHR Global Health Research Group on Atrial Fibrillation Management

**Affiliations:** a Institute of Applied Health Research, University of Birmingham, Birmingham, UK; b Faculdade de Medicina, Universidade, Sao Paulo, São Paulo, Brazil; c Center for Clinical and Epidemiologic Research and Division of Internal Medicine, University Hospital, University of São Paulo, São Paulo, Brazil; d Department of Cardiology, Chinese PLA General Hospital, Beijing, China; e Department of Community and Family Medicine, Faculty of Medicine, University of Jaffna, Jaffna, Sri Lanka; f Liverpool Centre for Cardiovascular Science, University of Liverpool, Liverpool, UK

**Keywords:** Early diagnosis, continuity of care, mixed-methods, cascade of care, pathways of care, Brazil, China, Sri Lanka

## Abstract

**Background:**

Identifying existing care pathways is the first step for understanding how services can be improved to enable early diagnosis and effective follow-up care for non-communicable diseases (NCDs); however, evidence on how care pathways can and should be identified in low- and middle-income countries (LMICs) is lacking.

**Objective:**

To describe generalisability and lessons learned from recruitment and data collection for the quantitative component of a mixed methods study designed to determine the care pathway for atrial fibrillation (AF) in Brazil, China and Sri Lanka.

**Methods:**

Adults (≥18 years) that spoke the local language and with an AF diagnosis were eligible. We excluded anyone with a hearing or cognitive impairment or ineligible address. Eligible participants were identified using electronic records in Brazil and China; in Sri Lanka, researchers attended the outpatient clinics to identify eligible participants. Data were collected using two quantitative questionnaires administered at least 2-months apart. A minimum sample size of 238 was required for each country.

**Results:**

The required sample size was met in Brazil (*n* = 267) and China (*n* = 298), but a large proportion of AF patients could not be contacted (47% and 27%, respectively) or refused to participate (36% and 38%, respectively). In Sri Lanka, recruitment was challenging, resulting in a reduced sample (*n* = 151). Mean age of participants from Brazil, China and Sri Lanka was 69 (SD = 11.3), 65 (SD = 12.8) and 58 (SD = 11.7), respectively. Females accounted for 49% of the Brazil sample, 62% in China and 70% in Sri Lanka.

**Conclusions:**

Generalisability was an issue in Brazil and China, as was selection bias. Recruitment bias was highlighted in Sri Lanka. Additional or alternative recruitment methods may be required to ensure generalisability and reduce bias in future studies aimed at identifying NCD care pathways in LMICs.

## Introduction

Non-communicable diseases (NCDs) contribute to 73% of global deaths, with stroke being the third leading cause [1]. However, there are substantial variations between low- and middle-income countries (LMICs) and high-income countries. Eighty-six percent of all stroke-related deaths and 89% of stroke-related disability-adjusted life years (DALYs) occur in LMICs [2]; the stroke mortality rate is 3.6 times higher in LMICs compared to high-income countries [2]. Healthcare professionals can and should identify existing conditions or risk factors that can be managed through medical or surgical interventions to prevent further complications or comorbidities. Primary healthcare units are best placed to provide screening tests and initial investigations of such conditions before referring the patient to a specialist for further investigation and treatment, but many LMICs suffer from low-resourced primary healthcare units with healthcare professionals untrained and unequipped to do so [3]. To know what elements of the health system needs further development and strengthening in LMICs to allow for early diagnoses and effective management of NCDs and risk factors, we must first understand current pathways of care for these conditions, including any barriers and facilitators for optimal care.

Atrial fibrillation (AF) is the most common arrhythmia in the world and a leading risk factor for stroke [4,5]. More than 37 million people had AF in 2017 [6], but with medication, AF can be well-controlled and the risk of stroke can be reduced by 64% [7]. In addition to stroke, AF is associated with a significantly increased risk of heart failure, myocardial infarction and all-cause mortality [8–12]. As the global population continues to age and risk factors become more prevalent, the global burden of AF is expected to increase by 66% over the next 30 years [6]. This projection does not account for the uncertainties around and probable underestimation of AF burden in LMICs where AF-related mortality and overall burden are likely to increase rapidly over the next decades [13].

### The optimal pathway of care for atrial fibrillation

The ABC (AF Better Care) pathway is an integrated approach that has been effectively adopted in many high-income countries to improve the management of AF [14]. This cost-effective [15] framework proposes to (A) avoid stroke through anticoagulation, (B) better manage symptoms with patient-centred decisions on rate or rhythm control, and (C) manage cardiovascular complications and other comorbidities with medical therapy and lifestyle changes [16]. For the first element of this framework (avoid stroke), five actions must be taken. First, AF should be diagnosed by identifying at-risk patients in primary care, thus, prior to stroke. Second, patients with suspected or confirmed AF should be referred to a specialised clinic or healthcare professional for confirming the diagnosis and ongoing monitoring and management. Third, the patient should be prescribed oral anticoagulants (OACs) based on an evidence-based risk assessment [17–20]. Fourth, the patient should adhere to the medications, and lastly, patients prescribed warfarin (the most common OAC in LMICs) should have monthly blood tests to ensure therapeutic range is met and to monitor the patient’s risk of blood clots and haemorrhaging [21]. If the therapeutic range is not met, warfarin dose should be adjusted accordingly.

Early diagnosis and continuity of follow-up care is essential for optimal AF care; however, significant barriers may exist for achieving this in LMICs and these barriers likely differ between countries and regions. In many LMICs, primary care utilisation may be poor, diagnostic equipment may be scarce and clinicians may lack knowledge on AF, associated risks and assessment tools [22]. Physical and financial access to clinics, medication and tests is an additional challenge in LMICs, including the lack of infrastructure necessary to collect blood samples and perform tests [23]. An issue not unique to LMICs is patient adherence to medication, tests and follow-up care which depend on patient and public awareness and understanding of AF, among other factors [24]. Whilst evidence suggest OAC use in LMICs is suboptimal [25,26], little is known about other aspects of the AF care pathway in LMICs.

### Methods for identifying pathways of care

There is a lack of evidence on how care pathways for NCDs should be investigated in LMICs. Selected studies have investigated the cascade of care for NCDs in LMICs [27,28]; however, such studies typically reveal the population-based prevalence of a condition, the proportion of those with the condition that have been diagnosed, the proportion of those diagnosed on medication and the proportion of those on medication having the condition under-control [29]. These studies are important for identifying where patients are lost within the cascade of care; though they often require large and expensive community-based cohort studies which are not always feasible [30]. Additionally, cascade of care statistics does not provide evidence on how and why patients are lost to care which is required for the development of targeted improvements to policy and practice and health system strengthening. On the contrary, pathways of care encompass the sequence of contacts that patients have with individuals and organisations for seeking and receiving healthcare [31]; this can highlight gaps and inefficacies for optimising care delivery.

Most high-income countries are now capable of using reliable and robust electronic records and data to understand NCD care pathways [32], but many limitations exist through the use of electronic records and is rarely an option for LMICs [33]. In LMICs, tuberculosis (TB) care pathways have been studied using the ‘patient pathway analysis’ method defined by Hanson et al [34] which relies on national survey data routinely collected by the World Health Organisation (WHO); however, these surveys collect limited data on NCDs [35]. Studies exist on the pathways of mental healthcare in LMICs; most use semi-structured interviews or the encounter form, developed by the WHO [36] specifically for understanding health-seeking behaviours in people suffering from psychoses [37,38]. Studies have also been conducted in LMICs to understand the pathway of care for children under five who have died [39], but this population is easily identifiable and the WHO verbal autopsy instrument is suitable for data collection with available training manuals and implementation support [40]. No existing tool exists for NCDs. Recruitment for TB and mental health patients can take place in TB clinics and mental health hospitals where a sufficient number of patients can be found [37,38]. For NCDs, designated clinics for specific conditions are often not available and a lack of electronic records can cause difficulties for identifying and recruiting patients with certain NCDs. Patients may also receive care from various facilities for their NCDs, more so than other conditions like TB which have designated clinics. The optimal way of identifying, recruiting and collecting data for determining NCD pathways of care is unknown, yet necessary for improving care for NCDs in LMICs.

### Study aims and objectives

We developed and conducted a prospective mixed-methods study designed to identify the AF care pathway regarding diagnosis and follow-up care in three diverse LMICs and to identify what the context-specific barriers and facilitators are for receiving an early diagnosis and high-quality continuity of care. This current paper aims to discuss the generalisability and lessons learned from the methods used in the quantitative component of the study to inform and improve future research aimed at identifying NCD care pathways in LMICs.

## Study design and methodology

### Study methods and tools

We developed and conducted a prospective mixed-methods study designed to identify the AF care pathway regarding diagnosis and follow-up care in three diverse LMICs and to identify what the context-specific barriers and facilitators are for receiving an early diagnosis and high-quality continuity of care; however, this paper focuses only on the quantitative component. Two questionnaires were developed and adapted from studies in Mongolia [41] and India [42] to collect quantitative data on AF diagnosis, management and care. Together, these questionnaires were designed to identify the pathway patients take to receive an AF diagnosis and AF follow-up care. The baseline questionnaire aimed to capture how and when an AF diagnosis was received whereas the follow-up questionnaire aimed to capture patients’ healthcare seeking behaviours and the care and medication received since baseline; the follow-up questionnaire was administered at least two months following the baseline questionnaire. The questionnaires were translated into Portuguese, Tamil, and Mandarin for Brazil, Sri Lanka and China, respectively, then back translated into English to check for accuracy. Before the study commenced, the questionnaires were piloted with ten patients in Brazil and four patients in Sri Lanka; no major changes resulted from piloting the tools.

### Settings

**Figure 1.**
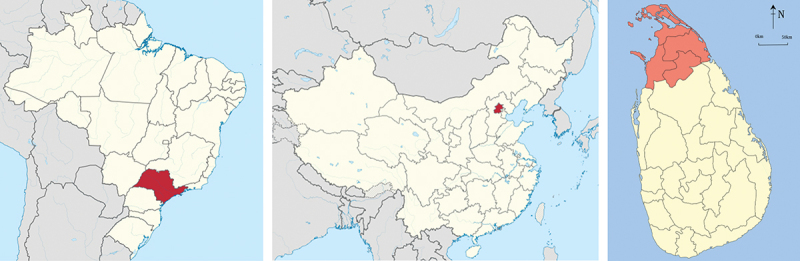
Map of each study site. From left to right and highlighted red: São Paulo, Brazil; Beijing, China; Northern Province, Sri Lanka.

This multi-centre study was carried out in two upper middle-income countries (Brazil and China) and one low-income country (Sri Lanka) [43]. Participants were recruited in the Butantan district of Brazil, an urban area of São Paulo (Figure 1) with an estimated prevalence of 2.4% for AF in adults aged 65 or older [44]. Medications and primary, secondary and tertiary care are free to all Brazilian citizens, including diagnostic tests, care and management of AF. There are 15 primary care units, one specialised cardiology clinic and one 258-bed community hospital in the Butantan district; however, patients were recruited from the 13 facilities that are associated with the University of São Paulo (the specialised cardiology clinic, community hospital and 11 primary care units).

Beijing was our study site in China (Figure 1), an urban and peri-urban area with more than 20 million people [45]. Most Chinese citizens are provided with health insurance; however, costs of and care covered by insurance plans vary across the country [46]. The PLA (People’s Liberation Army) General Hospital, a teaching and tertiary referral hospital, was the only facility that patients were recruited from in Beijing based on convenience sampling.

In Sri Lanka, patients were recruited from the Northern Province (Figure 1). The Northern Province is made up of five districts, including urban, rural and peri-urban settings, with an estimated population of 1.2 million people [47]. Healthcare is free in Sri Lanka; however, there is only one tertiary hospital in the Northern Province (Jaffna Teaching Hospital) with a cardiologist and equipment for conducting echocardiograms (ECHO) and measuring international normalised ratio (INR). AF patients were recruited from the Accident and Emergency (A&E) department, anticoagulation clinic and the medical clinic located at the Jaffna Teaching Hospital.

### Study participants and data collection

The inclusion criteria consisted of adults aged 18 or over that spoke Portuguese, Mandarin and Tamil in Brazil, China and Sri Lanka, respectively, and had a confirmed diagnosis of AF or an arrhythmia likely to be AF who had received care for AF from any of the included healthcare facilities. Patients were excluded if they had any hearing or cognitive impairment or if their home address was outside of the study site for each country.

In São Paulo, eligible participants were first identified from electronic records at each facility; they were then phoned for an invitation to participate. Two nurses trained in using the tools administered the questionnaires via phone or face-to-face using Google Forms between June 2019 and November 2020. At the PLA General Hospital in Beijing, eligible AF patients were identified by electronic records between July 2019 and July 2021. Trained nurses administered the questionnaires face-to-face or by phone, recorded responses on paper and entered the data into an electronic database within 24 h. Two people independently conducted checks of the electronic data entry for accuracy. Electronic records are routinely used and updated in the Brazil and China sites. In Jaffna, data was collected using KoBoCollect, an open-source software platform used in many LMICs [48]. Between October 2020 and March 2021, seven trained staff members with degrees in medicine or nursing approached patients whilst waiting in the anticoagulation and medical clinic to assess them for eligibility. Additionally, A&E staff notified the recruitment staff if any AF patients attended the A&E. The questionnaires were administered in person at the time eligibility was confirmed, following consent.

### Sample size calculation

To accurately estimate the proportion of AF patients diagnosed in primary care in an unknown population with ± 5% accuracy at the 95% confidence level (α = 0.05) a minimum sample size of 205 was required. This calculation assumed a 16% prevalence of AF patients being diagnosed in primary care based on baseline results from a cohort of more than 17,000 AF patients across 30 countries [49]. For administering the follow-up questionnaire, we increased the sample size by 15% to account for lost to follow up, resulting in a final sample size of 238 for each country.

## Recruitment results

**Figure 2.**
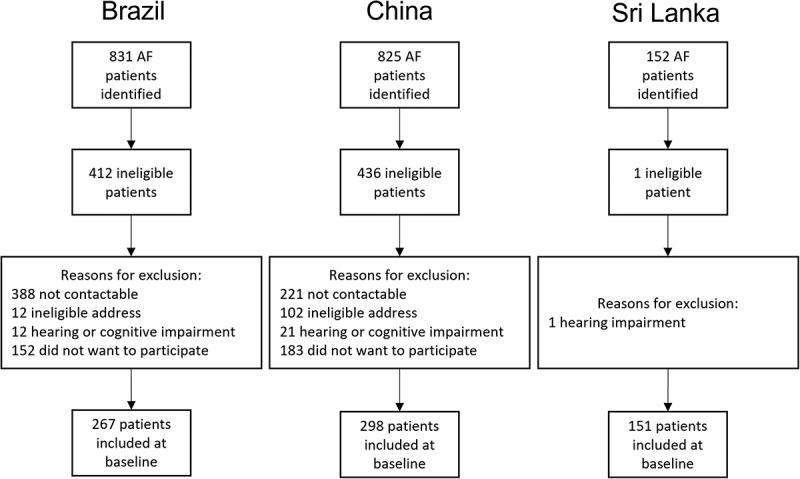
Recruitment flow chart for Brazil, China and Sri Lanka.

In São Paulo, 831 AF patients were identified from the 13 facilities; however, 388 were not contactable (Figure 2). Of those that were contacted (*n* = 443), 12 did not have an eligible address, 12 had a hearing or cognitive impairment and 152 were not interested in participating, leaving a total of 267 included in the study. Most data were collected over the phone (*n* = 231; 87%), but 13% (*n* = 36) of questionnaires were administered in person. The Beijing team identified 825 AF patients though 221 could not be contacted. Of the 604 remaining patients, 102 had an ineligible address, 21 had a hearing or cognitive impairment and 183 did not want to take part, leaving 298 AF patients in the final sample. Of these, 45 (15%) answered the questions over the phone and 253 (85%) answered them in person. In Jaffna, 152 AF patients were identified from clinics and the A&E department. One patient was not eligible due to a hearing impairment, leaving 151 AF patients included in the study; all agreed to take part.

## Participant characteristics

Mean age of participants from Brazil, China and Sri Lanka was 69 (standard deviation [SD] = 11.3), 65 (SD = 12.8) and 58 (SD = 11.7), respectively (Table 1). Females accounted for 49% of the sample in Brazil, 62% in China and 70% in Sri Lanka. Nearly all participants were married in the China sample (94%) whereas just over half were married in Brazil (53%) and Sri Lanka (62%). Many participants from the latter two countries were widowed (20% in Brazil and 21% in Sri Lanka). In Brazil, 51% of participants were White, 29% were Mixed race and 12% were Black. One hundred percent of participants in China identified as Han. Nearly all participants in Sri Lanka identified as Sri Lankan Tamil (97%) with others identifying as Moor (1%), Indian Tamil (1%) or Burgher (2%).

**Table 1.** Baseline demographic figures; N (%) unless otherwise stated.

	Brazil (*n* = 267)	China (*n* = 298)	Sri Lanka (*n* = 151)
Age			
Mean (SD)	69 (11.3)	65 (12.8)	58 (11.7)
<40	2 (0.7)	11 (3.7)	10 (6.6)
40 to 50	14 (5.2)	23 (7.7)	35 (23.2)
50 to 60	39 (14.6)	48 (16.1)	40 (26.5)
60 to 70	67 (25.1)	111 (37.2)	47 (31.1)
70 to 80	97 (36.3)	64 (21.5)	17 (11.3)
80 +	48 (18.0)	41 (13.8)	2 (1.3)
Missing/unknown	0 (0)	0 (0)	0 (0)
Gender			
Female	131 (49.1)	185 (62.1)	105 (69.5)
Male	136 (50.9)	113 (38.9)	46 (30.5)
Missing/unknown	0 (0)	0 (0)	0 (0)
Marital status			
Single	26 (9.7)	0 (0)	19 (12.6)
Married	140 (52.4)	279 (93.6)	94 (62.3)
Living with partner	20 (7.5)	1 (0.3)	0 (0)
Divorced	24 (9.0)	2 (0.7)	7 (4.6)
Widowed	51 (19.1)	5 (1.7)	31 (20.5)
Missing/unknown	6 (2.2)	11 (3.7)	0 (0)
Ethnicity^a^			
White	134 (50.2)	–	–
Black	31 (11.6)	–	–
Mixed race	76 (28.5)	–	–
Han	–	298 (100)	–
Sri Lankan Tamil	–	–	146 (96.7)
Moor	–	–	1 (0.7)
Indian Tamil	–	–	1 (0.7)
Burgher	–	–	3 (2.0)
Other	6 (2.2)	0 (0)	0 (0)
Missing/unknown	20 (7.5)	0 (0)	0 (0)
Education			
Did not complete primary school	86 (32.2)	18 (6.0)	22 (14.6)
Completed primary school	91 (34.1)	27 (9.1)	39 (25.8)
Completed secondary education	53 (19.9)	149 (50.0)	84 (55.6)
Holds undergraduate degree	25 (9.4)	98 (32.9)	6 (4.0)
Holds postgraduate degree	3 (1.1)	6 (2.0)	0 (0)
Missing/unknown	9 (3.4)	0 (0)	0 (0)
Literacy			
Illiterate	18 (6.7)	2 (0.7)	4 (2.6)
literate	249 (93.3)	296 (99.3)	147 (97.4)
Employment status			
Employed	44 (16.5)	63 (21.1)	28 (18.5)
Retired	182 (68.2)	211 (70.8)	12 (7.9)
Housewife	16 (6.0)	5 (1.7)	87 (57.6)
Student	1 (0.4)	2 (0.7)	0 (0)
Unable to work	20 (7.5)	9 (3.0)	20 (13.2)
Cannot find suitable job	3 (1.1)	1 (0.3)	1 (0.7)
Does not want to work	0 (0)	2 (0.7)	1 (0.7)
Other	1 (0.4)	5 (1.7)	2 (1.3)
Missing/unknown	0 (0)	0 (0)	0 (0)
Travel time to clinic			
Mean, minutes (SD)	54.2 (34.3)	174 (25.2)	51 (41.4)
Less than 30 minutes	53 (19.9)	69 (23.2)	69 (45.7)
Between 30 minutes and 1 hour	133 (49.8)	56 (18.8)	59 (39.1)
Between 1 and 2 hours	76 (28.5)	66 (22.1)	15 (9.9)
More than 2 hours	3 (1.1)	105 (35.2)	8 (5.3)
Missing/unknown	2 (0.7)	2 (0.7)	0 (0)
Mode of travel			
Walk	10 (3.7)	15 (5.0)	0 (0)
Bus or other public transit	149 (55.8)	177 (59.4)	80 (53.0)
Taxi	53 (19.9)	46 (15.4)	20 (13.2)
Family owned vehicle	53 (19.9)	35 (11.7)	33 (21.9)
Other	1 (0.4)	23 (7.7)	18 (11.9)
Missing/unknown	1 (0.4)	2 (0.7)	0 (0)
Comorbidities^b^			
Congestive heart failure	182 (68.2)	21 (7.0)	7 (4.6)
Hypertension	226 (84.6)	156 (52.3)	46 (30.5)
Blood clot	80 (30.0)	30 (10.1)	25 (16.6)
Peripheral vascular disease	72 (27.0)	76 (25.5)	0 (0)
Over function of thyroid gland	35 (13.1)	9 (3.0)	6 (4.0)
Long-term lung problems	23 (8.6)	21 (7.0)	24 (15.9)
Long-term kidney problems	30 (11.2)	7 (2.3)	7 (4.6)
Other	4 (1.5)	137 (46.0)	64 (42.4)
Missing/unknown	1 (0.4)	0 (0)	0 (0)

^a^Data is shown for ethnicity groups relevant to each country. A dash is presented where the ethnicity group is not relevant.

^b^Participants could choose more than one answer; percentages may not add up to 100%.

Education levels differed across each country with 32% of participants not completing primary school in Brazil versus 6% in China and 15% in Sri Lanka. The proportion of those that completed primary or secondary school was 54% in Brazil, 59% in China and 81% in Sri Lanka. Most participants were literate (93% in Brazil, 99% in China and 97% in Sri Lanka). In Brazil and China, most participants were retired (68% and 71%, respectively), whereas most participants were housewives in Sri Lanka (58%). Mean travel time to the AF clinic was less than an hour for participants in Brazil and Sri Lanka, (54 min [SD = 34] in Brazil and 51 min [SD = 41] in Sri Lanka); however, it was over 2 h for participants in China (174 min [SD = 25]).

A large proportion of participants from Brazil had comorbidities of congestive heart failure (68%) and hypertension (85%). The main co-existing conditions reported from the Chinese participants were hypertension (52%) and peripheral vascular disease (26%) whereas in Sri Lanka the frequency of comorbidities was much lower; the largest proportion of participants had hypertension (31%).

## Generlisability

The sex and ethnic characteristics of the Brazilian participants are in line with AF prevalent studies carried out in São Paulo [44,50,51]. Although participants in the current study were slightly older compared to two of these studies [50,51] and younger than participants from another study [44]. The prevalence of heart failure and hypertension were generally higher in the current study when compared to other studies of AF patients [44,50,52], including one study that also used self-reporting to identify conditions [52]. The differences in age and comorbidities could be due to the high proportion of eligible patients that were not contactable; it is plausible that the younger and healthier AF patients are more socially active or employed and less likely to be home and available. Conversely, age and prevalence of comorbidities reported by Chinese participants were in line with AF prevalent studies conducted in China [53–56]. The proportion of female participants in our China sample (62%) was higher compared to these formerly published studies (38 to 54%) [53–56] which could potentially be due to the high proportion of eligible patients that refused to participate; sex differences have been noted for non-participation in observational health studies with males more likely to decline due to time constraints [57]. These findings suggest the need for a more flexible schedule for research data collection to enable, younger, healthier and men, all more likely to work during daytime hours, to be recruited.

There are no AF prevalent studies to compare the demographics of the Sri Lankan participants to; however, we can cautiously make comparisons to population-based government statistics from Northern Province and AF data from neighbouring India. Marital status, education level and ethnicity data were in line with population-based statistics; however, the proportion of females was much higher in our study (70% compared to 51–53%) [47,58]. This was also the case compared to data from India where the GARFIELD-AF study reports 40% of females in their sample of AF patients recruited within six months following diagnosis [59]. This discrepancy could potentially be due to differences in healthcare seeking behaviours between females and males. We recruited AF patients that were currently receiving care which may indicate that females are more likely to maintain follow-up care/attend appointments following their AF diagnoses. This suggests that the Sri Lankan sample suffered from recruitment bias, with the presumption that AF patients not retained in care (who were omitted from our study site) systematically differ from those that are.

## Lessons learned

Availability of electronic health records in São Paulo and Beijing proved beneficial for identifying the required number of eligible participants. However, given the large proportion of eligible patients that could not be contacted or did not want to participate, sampling bias was likely introduced to both samples. Conversely, Identifying and recruiting eligible patients in Jaffna was a difficult task hence the inability to reach the required sample size. At the Jaffna Teaching Hospital, there was no dedicated day or place for anticoagulation management and electronic records are lacking. As a result, data collectors visited the clinics every day and waited long hours to identify and recruit eligible AF patients. A&E staff were asked to contact the research team if any AF patients came under their care; however, this occurred only twice. This lack of infrastructure in terms of electronic records and not having a designated day or place for AF patients to receive care likely contributed to the reduced sample size. However, AF is underdiagnosed in many LMICs [13], possibly leading to a lower than expected number of patients visiting the clinics in Jaffna. Together, these findings highlight the need for future care pathway studies in LMICs to closely consider time and resources required to recruit patients where electronic records are not available, and prevalence of the condition is unknown or potentially low; simultaneously it is important to understand that available electronic records in LMICs may not equate to a representative sample and an alternative or multiple recruitment strategies may be required.

Collection of data was mainly conducted over the phone in São Paulo due to a lack of private physical space to administer the questionnaires in person at any of the included healthcare facilities. Given the increase of mobile phone use in LMICs [60], this will increasingly be an appropriate way to collect data, particularly from patients with age-related NCDs where the population may be older with a high prevalence of comorbidities. Data collection over the phone may be preferred to reduce the need for patients to make unnecessary visits to healthcare facilities. However, patients in Jaffna were happy to use the long waiting times (1 to 2 h) to complete the questionnaires face-to-face. Long waiting times for healthcare appointments is a characteristic common in LMICs [61] and therefore could be utilised for data collection purposes in certain settings.

## Study limitations and strengths

This study included three structurally, culturally and socially different countries; thus, providing a major strength and opportunity to provide a discussion on the generalisability and lessons learned to inform future NCD pathway studies in LMICs. One limitation to mention is the overlap of recruitment and data collection with the COVID-19 pandemic. During the pandemic, services were changed and disrupted in all three countries. Partial or complete lockdowns and limitations on travel and healthcare provision occurred, likely impacting recruitment and data collection in ways impossible to identify. However, in São Paulo, recruitment and data collection was primarily completed via phone; therefore, we anticipate that the pandemic had limited impact on the Brazilian sample. Furthermore, visits to the hospital in Jaffna remained normal throughout the study period (i.e. during the pandemic) and all data collection was completed following arrangements to maintain social distancing. In Beijing, the time needed for recruitment and data collection was greatly impacted by the pandemic due to locally imposed restrictions that resulted in fewer patients attending hospital, though the required sample size was ultimately reached. Another limitation is the lack of data on why eligible patients refused to participate; such data could have aided our understanding of how our sample in Brazil and China was potentially biased.

## Conclusion

We describe the generalisability and lessons learned from recruitment and quantitative data collection of a prospective study in three LMICs (Brazil, China and Sri Lanka) for identifying the care pathway of AF, an NCD that requires early diagnosis and continuity of care to prevent morbidity and premature mortality. Whilst the tools used were the same across each setting, the methods for recruiting eligible participants and collecting data were by necessity locally adapted. However, our study highlights the need to consider sampling bias and additional recruitment methods to ensure generalisability in settings with electronic records. Conversely, in settings without electronic records, it is vital to understand the local epidemiology of the condition of interest, healthcare infrastructure and available resources before feasibility of using our methods can be ensured; if the condition being investigated is rare, then meeting the required sample size may be difficult and require extensive time and resources. These findings should guide future studies on how our methods can be adapted and improved upon for identifying care pathways for NCDs to understand where gaps may exist for providing effective management and care; thus, enabling improvements to be made to healthcare systems and services and ensuring life-saving continuity of care. This area of research will become increasingly important as the burden of NCDs, such as AF, continues to rise in LMICs.

## Paper context

The burden of non-communicable diseases (NCDs), including atrial fibrillation (AF) is increasing in low- and middle-income countries (LMICs), indicating the importance for understanding the patient care pathway for NCDs. Our study provides evidence on how the AF care pathway can be investigated using locally-adapted methods for recruitment and data collection in three LMICs. Future studies investigating similar NCD pathways should use our lessons learnt to reduce bias and ensure a representable, powered sample is achieved.
